# Sparse Data Analysis Strategy for Neural Spike Classification

**DOI:** 10.1155/2014/757068

**Published:** 2014-07-02

**Authors:** Vincent Vigneron, Hsin Chen

**Affiliations:** ^1^IBISC-Lab, Université d'Évry Val d'Essonne, 40 rue du Pelvoux, 91020 Courcouronnes, France; ^2^Department of Electrical Engineering, National Tsing Hua University, No. 101, Sec. 2, Kuang-Fu Road, Hsin-Chu 30013, Taiwan

## Abstract

Many of the multichannel extracellular recordings of neural activity consist of attempting to sort spikes on the basis of shared characteristics with some feature detection techniques. Then spikes can be sorted into distinct clusters. There are in general two main statistical issues: firstly, spike sorting can result in well-sorted units, but by with no means one can be sure that one is dealing with single units due to the number of neurons adjacent to the recording electrode. Secondly, the waveform dimensionality is reduced in a small subset of discriminating features. This shortening dimension effort was introduced as an aid to visualization and manual clustering, but also to reduce the computational complexity in automatic classification. We introduce a metric based on common neighbourhood to introduce sparsity in the dataset and separate data into more homogeneous subgroups. The approach is particularly well suited for clustering when the individual clusters are elongated (that is nonspherical). In addition it does need not to select the number of clusters, it is very efficient to visualize clusters in a dataset, it is robust to noise, it can handle imbalanced data, and it is fully automatic and deterministic.

## 1. Introduction

Neurophysiologists assume that the brain encodes information in the firing rate of neurons, that is, the number of “spikes” over a temporal interval. While many powerful imaging techniques have been used in neuroscience, extracellular recording remains the only choice that provides resolution of neuron activity in the brain. However, multiple extracellular recordings are useful only when the spikes generated by different neurons can be correctly sorted.

Lewicki [1] reviewed numerous methods that have been proposed to classify spikes. The usual assumptions for spike sorting are (1) that all spikes generated by a specific neuron are characterized by a similar waveform, (2) that this waveform is unique, and (3) that this waveform is conserved for each neuron during a stationary recording [2]. Analysis of neural recordings requires first detecting action potentials,* spikes*, from noise, which is achieved with thresholding discrimination by manual or semiautomatic classification methods. The second process is spikes sorting and produces a number of “spike trains” corresponding to the temporal sequence of real signals [3–5].

Among different methods used for spike sorting, template matching is one of the most popular procedures. The usual practice to produce templates is to use a “supervisor,” that is, an experienced and knowledgeable operator, to preliminarily classify the waveforms following a selection of templates corresponding to distinct neurons. Few methods have dealt with unsupervised template creation. Atiya [3] for instance used the Isodata clustering algorithm to estimate typical spike shapes and then compared all possible combinations of templates to find the combination with the highest likelihood. Letelier and Weber [6] applied Bayesian probability theory to quantify the probability of both the form and the number of spike shapes. Zouridakis and Tam [7] proposed a procedure based on fuzzy *k*-means clustering algorithms to create reliable spike templates. Some authors [8–10] used independent component analysis (ICA) for distinguishing the spikes according to their sources; the independence assumption of the firing neurons helps to identify spikes from the same source. In [11] the occurrence time information of spikes and features related to the shape simultaneously is applied to estimate the interspike interval for each neuron and sort the spikes using a Monte Carlo algorithm. Pouzat et al. [12] used an empirical characterization of the recording noise to optimize the action potentials clustering and for assessing the quality of each cluster. Zhang et al. [13] reconstructed the spike templates according to the clustering results from principal component analysis (PCA) and substractive clustering techniques. Probabilistic methods have been proposed [14, 15] and have focused on the modeling of each group in specific subspaces of low dimensionality.

Several approaches are associated with a visualization objective such as factorial methods [16, 17]. The latter methods can be* global* when they are based on the proximity between various groups such as graph methods or* local* when they evaluate the proximity between the individuals, like the hierarchical methods, they can also combine the local and global relations, as in the case of* seriation*.

A taxonomy of the methods was proposed by Carroll and Arabie [18] which associates a particular mode of seriation with each type of table.

**Figure 1:**
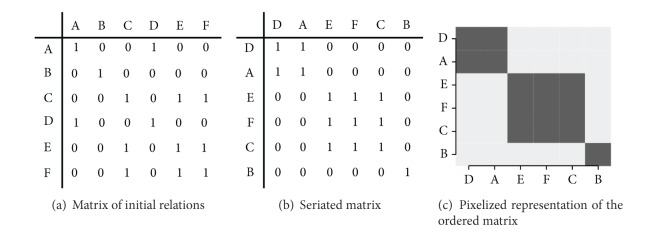
Effects of an algorithm of arrangement on a data set.

Seriation aims to display and to reveal natural clusters and their dependencies in a dataset only by reordering rows and columns so that the adjacent rows and, respectively, columns are the most similar. This situation is illustrated by Figure 1 where, starting from a table of relations presented by Figure 1(a), the lines and the columns permuted to form a partition in which the similar elements were gathered together, thus forming groups (Figure 1(b)), and, in order to better appreciate the presence of the diagonal structure per block, this ordered matrix is pixelized (Figure 1(c)). Such an approach could be connected with a local technique of ordered clustering in so far as information is brought on one hand about the local relations between individuals because of an order in the data and on the other hand about the total structure of the data. Seriation has other advantages outlined by several authors such as Arabie et al. [19], like the no need of prior knowledge on the number of clusters and direct visualization of the structure on the table of values.

These advantages might disappear when the data are noisy or* imbalanced* or when groups of data are superimposed. The presence of noisy data prevents a clear visualization of the various blocks and distinguishing the clusters becomes a difficult task. Our approach is based on symmetric binary matrices of similarities (or dissimilarities) linked to common neighborhood. Such matrices indicate similarities between pairs of observations and can be computed by different measures depending on the nature of the dataset such as Euclidean distances or more generally *p*-norm, correlation coefficients [20], or divergences [21] for example. A criterion derived from the problems of data compression selects the most compact ordered matrix—in the form of diagonal blocks—in order to obtain the most informative visualization off the intrinsic data structure. In some situations, too great a parsimony generates the ousting of underrepresented data forming very small clusters. To mitigate this nondetection, we propose a multiscale approach combining various levels of sparsity of the data.

This paper is organized in the following way: in Section 2, seriation is presented according to two different points of view, one as a mathematical optimization problem to be solved and the other on its algorithmic bases.  Section 3 details our original approach as well as a multiscale algorithm of the proposed arrangement, called Parsimonious Block-Clustering. Experiments on simulations and benchmark data are presented in Section 4.

## 2. Method

### 2.1. The Optimization Problem

Seriation seeks an order in the data that reveals the locality/proximity between adjacent lines or columns to thus reveal a structure. This order is obtained by successive permutations of lines and columns which makes it possible to tackle seriation optimization problem (The number of possible combinations of permutations of lines and of columns is *n*!*p*! for a rectangular *n* × *p* table or *n*! in the case of a symmetrical matrix of dissimilarity.) through two different angles: one being to determine all the best possible permutations, the other being related to the complexity of the solution (*np*-complete problem).

The seriation approach can be applied to any type of matrices but we focus in this work on dissimilarity matrices. Let us consider a set of *N* samples (*x*_1_,…, *x*_*N*_) described by a symmetrical matrix of dissimilarity *D* = (*d*_*ij*_)_*i*,*j*∈(1,…,*N*)_ of size *N* × *N* where each element *d*_*ij*_ gives a “measure” of dissimilarity between the pair of observations (*x*_*i*_, *x*_*j*_). Let Ψ define a permutation function which orders the elements of matrix *D*, according to a given criterion *C*. The objective of the seriation is thus to find the optimal permutation Ψ* which optimizes the arrangement criterion *C*, such that  

(1) $$\begin{array}{c} \Psi^{*}=\arg\underset{\Psi }{\max}C\left(\Psi \left(D\right)\right). \end{array}$$

These criteria are based on a measure of similarity *s*(·) between the successive elements of the matrix *D* and maximize max⁡∑_*i*=1_^*n*−1^ *s*(*i*, *i* + 1).

This measure of similarity is declined in a different way according to the authors as one can observe in Table 1. McCormick et al. [22] and Arabie and Hubert [23], for example, seek to maximize a* measure of effectiveness* (cf. *C*_6_ criterion in Table 1) based on the sum of the scalar products in lines and columns of the data matrix; this measure was generalized thereafter by Climer and Zhang [20]. Other authors, such as Hubert et al. [24] or Chen [25], based their optimization on the divergence measure between the matrix of dissimilarity and an anti-Robinson structure seeking to gather the values of the smallest dissimilarities around the diagonal (cf. *C*_4_ criterion). On the other hand, some authors such as Caraux and Pinloche [26] (cf. *C*_1_ and *C*_2_ criteria) or Brusco and Steinley [27] (cf. *C*_3_ criterion) rather seek to place the smallest dissimilarities out of the diagonal (Robinson structure). Lastly, in the framework of data compression, Johnson et al. [28] proposed to minimize a criterion based on the number of sequences of consecutive elements (on a line) different from 0 (cf. *C*_5_ criterion). Many authors proposed new criteria of arrangement like Niermann [29] who seeks to compare each observation with its adjacent neighbors through vicinity criteria (cf. *C*_7_ criterion) or Batagelj [30] or Doreian et al. [31] who propose criteria of structural equivalence or Dhillon et al. [32] who use mutual information and an entropy-based criterion.

These recent approaches require a* prior* knowledge of the number of clusters formed by the individuals and the variables whose determination is not trivial.

### 2.2. A Family of Embedded Binary Matrices

To deal with the problem of imbalanced datasets, noisy data, overlapping clusters, and outliers, we propose a new algorithm based on a family of embedded binary matrices which stands for different degrees of sparsity of the data. The binary matrices are ordered according to an algorithm named Parsimonious Block-Clustering (PB-clus). This algorithm makes it possible to select the level of parsimony to produce the optimal compact block structure.

In our approach, the degree of vicinity is defined as a “threshold value” equal to the number of common neighbors between pairs of observations after which pairs of observations are eliminated. The larger the number of common neighbours imposed is, the more parsimonious the matrix will be (filled with zeros). Hence, the degree of parsimony is associated with the degree of common vicinity. Let us consider a data matrix *X* with elements in *R* ^*p*^ and *X* ^*d*^ = (*x*_*ij*_^*d*^), *i*, *j* ∈ {1,…, *n*} the dissimilarity matrix associated to *X*, the choice of the distance function depending on the type of data: it can be an Euclidean distance between individuals *i* and *j* (and more generally *p*-norm), a correlation, or any other function characterizing the concept of proximity between pairs of observations (see Table 1). Let *A* = (*a*_*ij*_), *i*, *j* ∈ {1,…, *n*}, and the (0,1)-matrix with elements

(2) $$\begin{array}{c} a_{ij}=\{\begin{array}{cc} 1 & \text{if}\;\;x_{ij}^{d}\leq \epsilon \;\\ 0 & \text{if}\;\;x_{ij}^{d}>\epsilon , \end{array} \end{array}$$

where *ϵ* is the threshold characterizing the proximity of the pairs of observations. Its value can be given arbitrarily; we propose to fix it at the first quartile of the distribution of the distances between pairs of observations. In addition, the matrix of similarity is symmetrical; that is, *a*_*ij*_ = *a*_*ji*_. Let the Gram matrix *B* = *A* ^*T*^ *A* where each element *b*_*ij*_ is the number of neighbors of the two data *i* and *j*. This matrix corresponds to a matrix of common vicinity.

**Definition 1 .**

A binary matrix *B*_*λ*_*m*__ = (*b*_*ij*_^*λ*_*m*_^), *i*, *j* ∈ {1,…, *n*}, parsimonious with a degree *λ*_*m*_ (with *m* ∈ {1,…, *M*}) is characterized by

(3) $$\begin{array}{c} b_{ij}^{\lambda_{m}}=\{\begin{array}{cc} 1 & \text{if}\;\;b_{ij}\geq \epsilon \\ 0 & \text{if}\;\;b_{ij}<\epsilon , \end{array} \end{array}$$

where *b*_*ij*_ represent the elements of the Gram matrix *B* defined previously. The set (*B*_*λ*_*m*__,…, *B*_*λ*_*M*__, ) forms a family of binary matrices whose level of parsimony is related to the number of common neighbors.

Taking into consideration this definition, the greater *λ*_*m*_ the fewer the number of pairs of observations which satisfy this condition. The associated matrix will contain a greater number of zeros and will thus be more parsimonious. The sequence (*λ*_*m*_)  *m* ∈ {1,…, *M*} such that *λ*_1_ < ⋯<*λ*_*M*_ makes it possible to establish an order relation ⊂ between the *M* elements of the set *B*_*λ*_*m*__ *m* ∈ {1,…, *M*}:

(4) $$\begin{array}{c} B_{\lambda_{M}}\subset B_{\lambda_{M-1}}\subset \cdots \subset B_{\lambda_{1}}, \end{array}$$

in which the most parsimonious matrix is contained in all the other matrices of its family. One of the advantages of such a matrix is the cancellation of the extreme values and of the noise when the level of parsimony increases, which facilitates the arrangement of the matrix as well as the appearance of adiagonal block structure. In relation to this family of matrices, a question remains: how to obtain the “best” level of parsimony, that is, the one which will make it possible to obtain a comprehensive visualization of the data structure?

The ordered matrix *B*_*λ*_*m*_,ord_* = (*b*_*ij*,ord_^*λ*_*m*_^)_ord_, *i*, *j* ∈ {1,…, *n*}, *m* ∈ *I* with the set *I* ∈ {1,…, *M*} contained in a set of ordered matrices, verifies that

(5) $$\begin{array}{c} {\text{B}}_{\lambda_{m},\text{ord}}^{*}=\arg\underset{m\in I}{\min}C_{\lambda_{m}}=\arg\underset{m\in I}{\min}\overset{n-2}{\underset{i=1}{\sum }}\overset{n-1}{\underset{j=i+1}{\sum }}\frac{\left|b_{ij,\text{ord}}^{\lambda_{m}}-b_{i(j+1),\text{ord}}^{\lambda_{m}}\right|}{\left|b_{ij}^{\lambda_{m}}-b_{i(j+1)}^{\lambda_{m}}\right|}. \end{array}$$

This criterion is based on the idea that the fewer the alternations between the 0 and the 1 on the lines of the matrix considered, the more compact a structure this matrix will have. Indeed, in Table 2, if one considers the quantity ∑_*i*=1_^*n*^∑_*j*=1_^*n*−1^|*b*_*ij*,ord_^*λ*_*m*_^ − *b*_*i*(*j*+1),ord_^*λ*_*m*_^| accounting for the number of changes between the 0 and the 1 of an ordered matrix of degree *λ*_*m*_ and the quantity |*b*_*ij*,ord_^*λ*_*m*_^ − *b*_*i*(*j*+1),ord_^*λ*_*m*_^| associated with the nonarranged matrix of the same degree, it is notable that the number of changes between the 0 and the 1 stays smaller in the case of the ordered matrices. As the degree of parsimony increases, the number of alternations between the 0 and the 1 falls: in the example, the numerator ∑_*i*=1_^*n*^∑_*j*=1_^*n*−1^|*b*_*ij*,ord_^*λ*_*m*_^ − *b*_*i*(*j*+1),ord_^*λ*_*m*_^| is equal to 9 for a level *λ* = 1 and to 3 when the degree of parsimony is 3. In order for the selection criteria not to be biased in favour of an infinite sparsity, *C*_*λ*_*m*__ is standardized by the number of alternations between the 0 and the 1 of the nonordered binary matrix associated with the same degree of parsimony. Thus, according to the example of Table 2, the level of parsimony retained is *λ* ≥ 2.

Let us note that, at this level, a structure with two groups is selected and a piece of data that can be regarded as extreme data is excluded. This criterion derives from the concept of* run* used in* data compression* [28, 33], characterizing the biggest sequences of 1 on a line in a Boolean matrix. The chosen criterion *C*_*λ*_*m*__ is related to the full number of changes present in the nonordered binary matrix of the same degree of parsimony so that it is not skewed in favour of an infinite parsimony or conversely, of too low a parsimony.

### 2.3. The Pb-Clus Geometry-Based Criterion

There are a plethora of criteria for the task of seriation [34] but the reordering algorithm that we proposed is based on the inner product because of its geometric interpretation. Since our work is based on symmetric matrices, the Tanimoto's norm (is also based on the dot product but adapted for binary data.) defined by *x*_*i*_^*T*^ *x*_*j*_/(*x*_*i*_^*T*^ *x*_*i*_ + *x*_*j*_^*T*^ *x*_*j*_ − *x*_*j*_^*T*^ *x*_*i*_) can be used for binary matrices *B* ^*λ*_*m*_^ of parsimony degrees *λ*_*m*_  (∀*m* ∈ {1,…, *M*}) defined in Section 2.2.

The permutation function Ψ which seeks to optimize the sum of the consecutive scalars can be written as

(6) $$\begin{array}{c} \Psi^{*}=\arg\underset{\Psi }{\max}\overset{n-1}{\underset{i=1}{\sum }}\frac{{b_{\Psi (i)}^{\lambda_{m}}}^{T}b_{\Psi (i+1)}^{\lambda_{m}}}{||b_{\Psi (i)}^{\lambda_{m}}||+||b_{\Psi (i+1)}^{\lambda_{m}}||-{b_{\Psi (i)}^{\lambda_{m}}}^{T}b_{\Psi (i+1)}^{\lambda_{m}}}. \end{array}$$

This criterion is based on the principle of* connected components*: when several observations share the same neighborhood then these observations will belong to the same cluster or to the nearest clusters. The algorithm is based on a branch and bound method meaning that an exhaustive search is made in various subsets that are determined by the geometric properties of the dot product: the algorithm first searches the independent vectors which the separated clusters produce, then considers the connected component of each of these vectors and finally, and reorders the correlated vectors in each group. These steps can be done for a binary neighborhood matrix *B*_*λ*_ with level *λ* in the following way.

1. Compute a matrix of dot products (inner products or Tanimoto's product) for each pair of columns of *X*_*λ*_ without considering the columns full of zeros.
2. Select a column and find its connected components. Then find an orthogonal vector of the previous column and extract its connected components. This procedure is performed until there are no more vectors. In this way, several independent submatrices are built.
3. In each submatrix, place the most correlated vector alongside the first column and keep on doing this process until the submatrix is reordered.
4. Gather the rearranged submatrices and apply this order to *B*_*λ*_.

The most informative visualization in terms of block-matrix is derived from the concept of* run* in compression approaches which characterizes a maximal sequences of nonzero entries in a row of a Boolean matrix [33]. It is intuitive that the fewer changes between series of ones and zeros are on each row the better the reordered matrix is. Since the sizes of the binary neighborhood matrices are different, this quantity is normalized by the minimum between the number of zeros or the number of ones of each rows so that

(7) $$\begin{array}{c} C_{\lambda }=\overset{n_{\lambda }}{\underset{i=1}{\sum }}\frac{\text{car}{\text{d}}_{i}\left(0,1\right)+\text{car}{\text{d}}_{i}\left(1,0\right)}{\min\left(\text{car}{\text{d}}_{i}\left(0,0\right),\text{car}{\text{d}}_{i}\left(1,1\right)\right)}, \end{array}$$

where *n*_*λ*_ is the number of nonzero columns of the reordered matrix *B*_*λ*_.

The algorithm enables us to find all the connected components of a cluster and to display relationships between clusters. This algorithm is straight forward deterministic algorithm, meaning that for a current move, the previous permutations are not challenged. Such an approach does not pretend to be optimal compared with the other approaches proposed in the literature but remains efficient and very fast even for large datasets and performs well when the data are noisy.

Since the proposed algorithm is a forward procedure (see Table 1), the final rearrangement obtained depends strongly on the first column selected in each submatrix. To deal with this problem, we propose to select a central observation for each submatrix to initialize the algorithm. The initialization is based on the idea that if we find a central observation in each cluster, then all connected components can be gathered. So, the first column is selected according to the number of strong correlated vectors which has to be maximum.

Lastly, Pb-Clus has a higher cost of calculation than the other methods of seriation since the arrangement is carried out not on only one matrix but on *M* matrices relative to different degrees of parsimony. In the case of a matrix of size *n* × *n* with *K* groups of same size *n*/*K*, there are at most *K*(*n*/*K*)! calculations. As the degree of parsimony increases, the matrix is filled with columns (lines) of zeros, which decreases the number of elements to be arranged, and consequently the computing time. The calculation cost would remain significantly lower than *M* · *K*(*n*/*K*)!.

## 3. Experiments on Simulated Data

### 3.1. Case of Non-Separated Clusters

**Figure 2:**
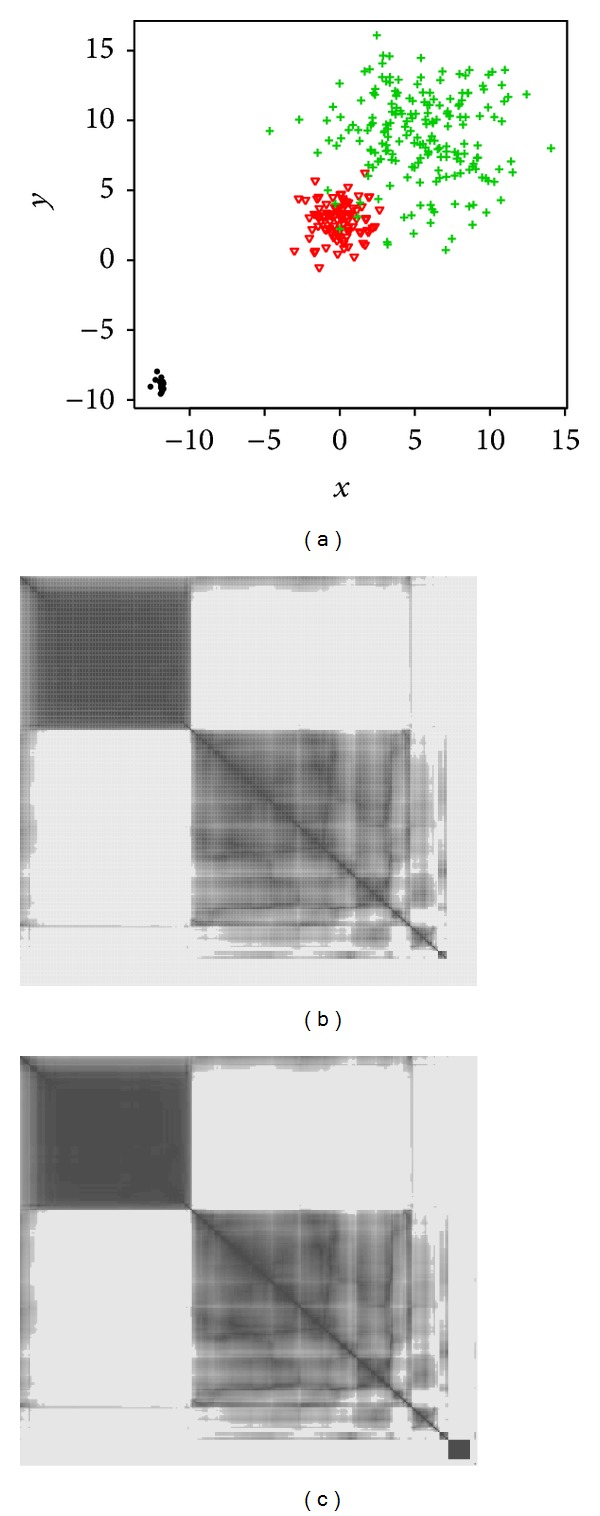
(a) Projection of the data in their space (b) visualizations of the central matrix ordered by Pb-Clus with a sparsity level of 16 common neighbors and (c) 8 common neighbors.

For this experiment, the data are simulated from three different 2-dimensional Gaussian mixtures with large variances and two clusters are superposed as illustrated in Figure 2(a). The first cluster is formed of 5% of the data (15 observations) while the two others account for 32% and 63% of the data, respectively (i.e., 100 and 200 observations). The central partition linked to this situation is represented in Figure 2(b) with a sparsity threshold of 16 common neighbors.

For this level of parsimony, more than 6% of the data were excluded which results in the removal of the smallest cluster. For a level of 8 common neighbors, it is possible to recover the third cluster.

Even if the central visualization Figure 2(c) is a bit less clear than previously, it is still informative and three different clusters can be seen. Moreover, the superposition of two clusters can be identified since in the central visualization, the two relative squares are inscribed in a bigger square which means correlations or proximities between these two groups. Lastly, among the seriated data, 98% have been correctly classified.

### 3.2. Influence of the Level of Superimposition of Clusters

In this second experiment, we seek to evaluate the influence of the level of covering of clusters in the search for a data structure. With this intention, we simulated 3 Gaussian distributions in a 2-dimensional space so that their respective averages check: *m*_1_ = (*x*,*y*)^*T*^,  *m*_2_ = (*x*,−*y*)^*T*^,  *m*_3_ = (0,−*y*)^*T*^ with *y* ∈ [0,0.3], and *y* ∈ [0,0.225]. Consequently, the relative position of the averages varies and this variation determines the level of superposition of the groups. Thus, when *x* = 0 and *y* = 0, the 3 groups are mixed and that corresponds to a superposition of 100%. In the opposite case of separate groups where the covering rate is zero, the averages of the clusters check: *m*_1_ = (0.3,0.225)^*T*^,  *m*_2_ = (0.3,−0.225)^*T*^,  *m*_3_ = (0,−0.225)^*T*^.  Table 3 presents the evolution of the sparsity level and its associated ousting rate, according to the covering of the groups.

First of all, one notices that the greater the superposition of the clusters is the more the *C*_*λ*_ criterion selects a parsimonious representation of the data. Indeed, when the visible data structure becomes less marked, this effect is balanced by a greater sparsity in the data with a bigger common vicinity. In the same way, as the data structure becomes increasingly complex, the rate of classification related to the subsets of seriated data decreases as well as the quality of visualization. In our example, beyond a rate of covering of the data of 40%, the rate of classification becomes weak (<60%) since the algorithm Pb-Clus no longer detects a structure in the data and this, whatever the level of parsimony imposed.

### 3.3. Case of Noisy Data

**Figure 3:**
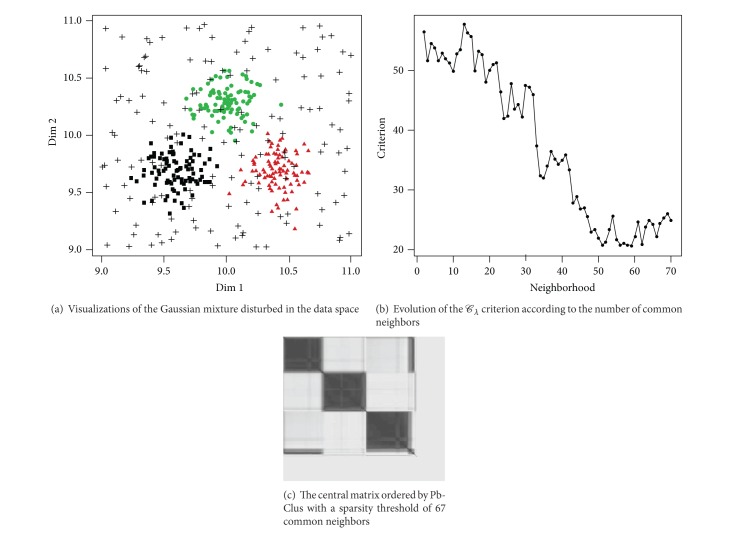
Seriation in the case of noisy data.

**Figure 4:**
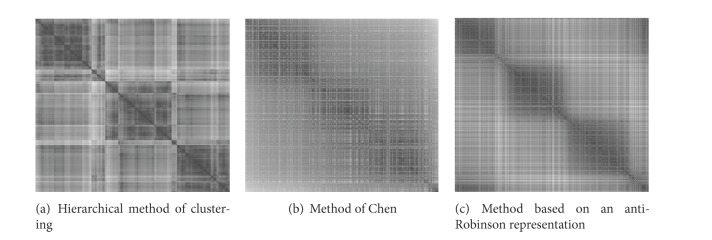
Visualizations of the pixelized distance matrix seriated.

In this experiment, 30% of the data are replaced by a uniform noise in a hypercube [−1,1]^4^ and the rest of the data are distributed from a mixture of three closed four dimensional Gaussian distributions as illustrated in Figure 3(a).  Figure 4(c) depicts the central visualization which brings out a natural structure of three clusters in the dataset even if the data are noisy.

Figure 3(b) presents the evolution of the compactness criterion *S*_*λ*_ according to the various degrees of parsimony, namely, the number of common neighbors. The central partition (Figure 3(c)) selected is the one for which the *C*_*λ*_ criterion is minimal. This corresponds to a common vicinity of 59. This sparsity results in the ousting of 16% of the data and only 84% of the initial data make it possible to obtain a block diagonal representation; the subsets of excluded data are entirely made of noisy data. The rate of correct classification among the seriated data amounts to 99%, which implies that these subsets of seriated data are a structural visualization of the 3 clusters. In order to evaluate the performance of our approach, three methods of seriation based on distance matrices were applied: hierarchical clustering (HC) for the seriation (Figure 4(a)), the approach of Chen based on an anti-Robinson structure [25] (Figure 4(b)), and another method of anti-Robinson seriation by simulated annealing [35] (Figure 4(c)).

Among the methods of seriation used, we notice that only the central partition provided by Pb-Clus brings a clear visualization of the three clusters. The representation of this structure in three distinct groups is possible thanks to the family of parsimonious binary matrices. Indeed, the higher the degree of parsimony in the matrices, the greater the decrease in the quantity of noisy data taken into account.

### 3.4. Influence of the Noise Level

**Figure 5:**
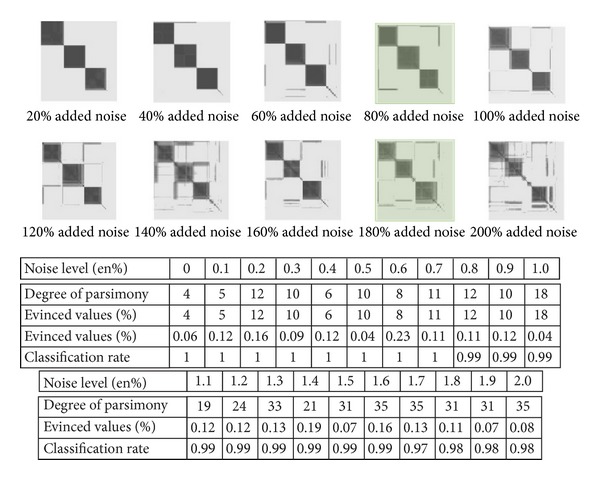
Visualization of the data structures according to various levels of noise added to the initial data.

This second experiment aims to demonstrate the behavior of Pb-Clus in the case of very noisy data. For this purpose, we simulated three 2-dimensional Gaussian distributions of 50 observations each with the following means *m*_1_ = (−0.4,−0.3)^*T*^, *m*_2_ = (−0.4,−0.3)^*T*^, and *m*_3_ = (0,0.3)^*T*^, respectively, and matrix of variance-covariance *S* = diag⁡(0.1,0.1). These groups are voluntarily separated in order to be able to evaluate the sensitivity of the algorithm to the noise. The noisy data were generated according to a uniform law on the support [−1,1]^2^. To evaluate the impact of the noise on visualization, we varied the quantity of noise from 10% to 200% of the number of data in the initial sample.  Figure 5 presents how the visualization of the data evolves with additional noise.

One notes that the group visualization degrades little with the additional noise. Indeed, in Figure 5, the structure is degraded only when the disturbed data represent more than half of the whole data.

### 3.5. Comparison on Classical Datasets

In this section, we compare the performance of PB Clus in terms of visualization firstly with two other methods of seriation, one using hierarchical classification (HC) and the other using a criterion of divergence related to an anti-Robinson structure described in Hahsler et al. [36] and, secondly, with an unsupervised classification method based on the Euclidean distances, the *k*-means. The 5 chosen datasets are detailed below.

- Fisher's irises database collects 3 different species of iris in the Gaspé peninsula:* setosa, virginica* and the* versicolor*. Each species is represented by 50 flowers which are described by 4 morphometric characteristics based on the width and the length of their sepals and their petals. This database is extremely popular in the statistical community because of difficulty of distinguishing the virginica and the versicolor.
- The* ruspini* data come from work of Ruspini [37] on clustering: they are made of 75 points in 2 dimensions and divided into 4 homogeneous and balanced classes.
- The* townships* data are binary data reporting the presence or the absence of 9 descriptive characteristics of 16 cities, such as the presence or the absence of universities, agricultural cooperatives, and railroads. There is no information on the number of groups structuring the data.
- *Old Faithful geyser data* evaluate the time between two eruptions of geysers of the national park of Yellowstone of Wyoming (USA) and their duration. They are characterized by 272 observations [38].
- The* geysers* data represent a full version of the preceding data that were collected by Azzalini and Bowman [39]. These relate to the 299 eruptions which were studied (same types of measurements as previously) between 1st and 15th August, 1985.

The quality of the visualization is calculated from two criteria proposed by Niermann [29] and presented in Section 2; the partition obtained will be evaluated by cross-validation with the true label when available or with the labels estimated by the *k*-means. As the latter supposes a prior knowledge of the number of groups of the mixture, we use the number of clusters detected by Pb-Clus in order to obtain comparable partitions.

**Figure 6:**
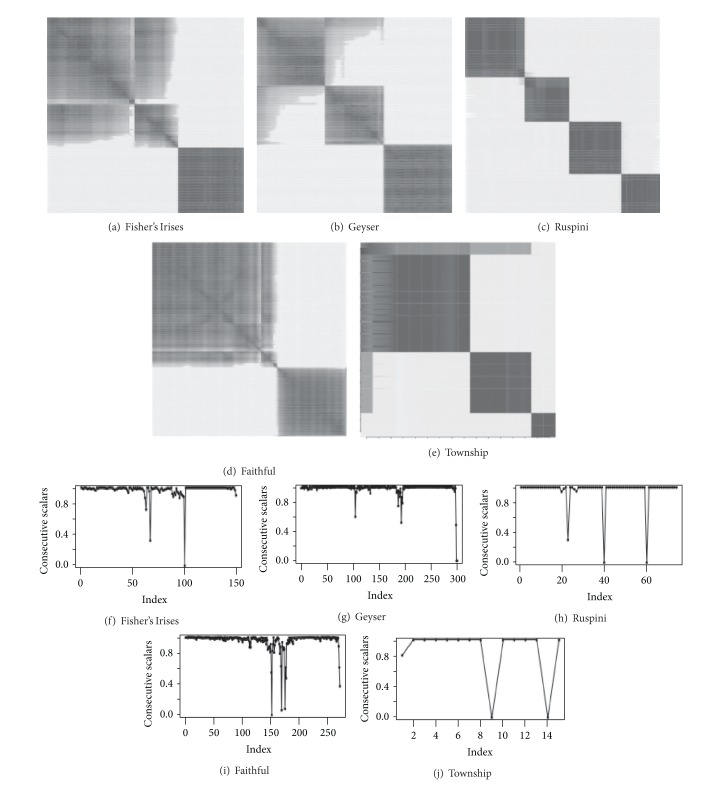
(a)–(e) rearranged matrices obtained with the PB-Clust (f)–(j) consecutive scalars resulting from the rearranged matrices.

The right-hand column of Figure 6 represents the consecutive dot products of elements *i* and *i* + 1 ordered out of the 5 previous databases. These curves of consecutive dot product give an evaluation of the proximity between two adjacent observations and points of rupture for the passage of one cluster to another, which makes it possible to select the number of clusters in the mixture and to obtain a partition of the data. In Figure 6 the left-hand column of represents the central visualization of the parsimonious matrix ordered with the algorithm PB-Clus. In the case of the Fisher's irises, the observation of its central matrix of degree of common vicinity 8 shows a total structure of two clusters.

One finds here the particular structure of the irises in which the* versicolor* and the* virginica* are not very distinct species. In addition, this partition in 3 groups is confirmed by the 2 break points present on the curves of its consecutive dot products. These 2 graphs demonstrate the performance of our parsimonious approach for the visualization of the data, especially as the methods of clustering which select one optimal model with 3 iris classes are rare (cf. mixture models of Raftery and Dean [40]). In the case of the* Ruspini* and the* Old Faithful* data, ruptures on the curve of the consecutive dot products are clear and large which show the total disconnection of the clusters between them. The same conclusion is visible on their ordered central matrix of degree 5 for the* Ruspini* data and of degree 2 for the* Faithful* data.

On the contrary, the* Geysers* and the* Townships* data present small breaking points. In the case of* Geysers* data, they are explained by the proximity of the clusters. Then, in the case of the* Townships* data, the curve of the consecutive scalars shows that the first city is, certainly, connected to the 7 following cities but less strongly than these 7 cities between each other. The central visualization of the parsimonious ordered matrix of degree 2 with Pb-Clus brings a better comprehension of the relationships between the cities. Indeed, it is noticed that the first data is strongly correlated with two distinct blocks of cities. This is confirmed by an analysis of Hahsler et al. who showed the existence of a structure with 3 groups: urban cities, country towns, and transition cities. This first evaluation based on our visual perception is supplemented by the measure of quality based on seriation criteria evaluating the vicinity in the ordered matrix.  Table 4 evaluates the performances of 3 methods of seriation, the best method being the one whose criterion is minimum. It is noticed that the 2 criteria of Niermann are minimum for a parsimonious approach for all the databases.

Lastly, Table 5 presents the tables of cross-classification with the true label for the irises of Fisher and with the labels obtained by *k*-means in the case of the data* Ruspini, Townships, Geysers, and Faithful*. Let us note that in the case of the* irises* and* Geysers* data, we threshold the scalars in order to obtain a label for each data. Concerning the Fisher irises, the correct classification rate of PB-Clus is 89.0%, slightly weaker than that obtained by the *k*-means (90.6%). This difference in rate is related to the data located at the intersection of the virginica and the versicolor and with initialization of our algorithm. For the other data files, one observes that the partitions obtained by Pb-Clus and the *k*-means agree almost perfectly, the rates of classification bordering 98%.

## 4. Experimental Methods

In this section, we approach the task of classifying spike waveforms using PB-Clus.

### 4.1. Animal Training and Behavioral Tasks

The detection of neural spike activity is a technical challenge that is a prerequisite for studying many types of brain function (for more details see Vigneron et al. [41]).

The study, approved by the Institutional Animal Care and Use Committee at the National Chiao Tung University, was conducted according to the standards established in the Guide for the Care and Use of Laboratory Animals. Four male rats weighing 250–300 g (BioLASCO Taiwan Corp., Ltd.) were individually housed applying a 12 h light/dark cycle, with access to food and water* ad libitum*.

Dataset was collected from the motor cortex of awake animals performing a simple reward task. In this task, male rats (BioLACO Taiwan Co.,Ltd) were trained to press a lever to initiate a trial in return for a water reward. The animals were water restricted 8-hours/day during training and recording session but food was always provided to the animal ad lib every day.

### 4.2. Chronic Animal Preparation and Neural Ensemble Recording

The animals were anesthetized with pentobarbital (50 mg/kg i.p.) and placed on a standard stereotaxic apparatus (Model 9000, David Kopf, USA). The dura was retracted carefully before the electrode array was implanted. The pairs of 8 microwire electrode arrays (no.15140/13848, 50 m in diameter; California Fine Wire Co., USA) were implanted into the layer V of the primary motor cortex (M1). The area related to forelimb movement is located anterior 2–4 mm and lateral 2–4 mm from bregma. After implantation, the exposed brain should be sealed with dental acrylic and a recovery time of a week is needed.

**Figure 7:**
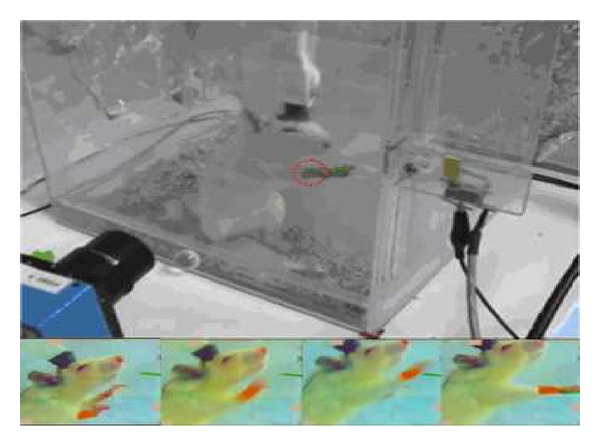
The experimental setup (top). Light-color (red virtual ring) was belted up the right forelimb to be recognized the trajectory by video tracking system. The sequence images captured the rat performing the lever press tasks in return for a reward of water drinking (bottom).

During the recording sessions, the animal was free to move within the behavior task box (30 cm × 30 cm × 60 cm), where rats only pressed the lever via the right forelimb, and then they received 1-mL water reward as shown in Figure 7. A multichannel Acquisition Processor (MAP, Plexon Inc., USA) was used to record neural signals. The recorded neural signals were transmitted from the head-stage to an amplifier, through a band-pass filter (spike preamp filter: 450–5 kHz; gain: 15,000–20,000), and sampled at 40 kHz per channel. Simultaneously, the animal's behavior was recorded by the video tracking system (CinePlex, Plexon Inc., USA) and examined to ensure that it was consistent for all trials included in a given analysis.

### 4.3. Preprocessing

Neural activity was collected from 400–700 ms before to 200–300 ms after lever release for each trail. Action potentials (spikes) crossing set thresholds were detected and sorted and the firing rate for each neuron was computed in 33 ms time bins. Since the signals are collected with 10 nanometers invasive probes, the noise effects are limited.

The experiment was made on 16 channels which collected EEG signals from microprobes which are implanted in the layer V of the M1 region of a rat.

### 4.4. Manual Scatterplot Classification

A method for classification is by plotting a selection of 2 or 3 spike features in a scatter diagram. This results in a 2- or 3-D graph with separate groups. The groups can only be assigned when there is enough spacing between the groups. Elliptic shaped areas are drawn around the groups isolating the classes.

### 4.5. Spike Waveforms Classification

To both reduce the size of these patterns and to cluster the spike mixture in a finite number of classes, we use two different tools: a seriation approach (PB-Clus) and a subspace clustering approach [42], named MDA (Mixture Discriminant analysis). Statistical discriminant analysis methods such as MDA aims to find both a parsimonious and discriminative fit for the data in order to ease the clustering and the visualization of the clustered data in a Gaussian mixture model context. MDA, developed by Hastie and Tibshirani [43], is a generalization of LDA (Linear Discriminant Analysis) in which each class is modeled by a mixture of Gaussians (see [44, chp. 4] for more details). This modelization gives more flexibility in the classification rule than LDA and allows MDA to take into account heterogeneity in a class. Breiman et al. [45], MacLachlan and Basford [46] have actually contributed and tested this generative approach on many fields. This latent subspace orientation is chosen such as it best discriminates the groups. The quality of the partition obtained by both approaches will be measured by the Fisher index which is defined by the ratio between the within (*S*_*w*_) and the between (*S*_*B*_) scatter matrices:

(8) $$\begin{array}{c} F_{\text{index}}=\frac{S_{w}}{S_{B}}=\frac{\sum_{k=1}^{K}\sum_{i\in C_{k}}^{\;}\left(x_{i}-m_{k}\right){\left(x_{i}-m_{k}\right)}^{t}}{\sum_{k=1}^{K}n_{k}\left(m_{k}-\bar{x}\right){\left(m_{k}-\bar{x}\right)}^{t}}, \end{array}$$

where *m*_*k*_ = (1/*n*_*k*_)∑_*i*∈*C*_*k*__^*K*^ *x*_*i*_ is the empirical mean of the observed column vector *x*_*i*_ in the class *k* and $\bar{x}=\left(1/n\right)\sum_{k=1}^{K}n_{k}m_{k}$ is the mean column vector of the observations. Besides, both methods will be compared with a traditional approach of clustering which first reduces the dimension by principal component analysis (PCA) and then clusters the data in the projected space and refers in this paper to PCA-EM. Clustering accuracy will be computed between the partition obtained by both approaches and that obtained by a *k*-means approach.

### 4.6. Results for Some Prominent Channels

This first study aims to satisfy the existence of 4 classes of spikes. For this experiment, the clustering task was made channel by channel and, in each channel, we consider all the different events which correspond to movements of the rat. Finally, for each event, many spikes were recorded. Each normalized spike waveform is a time series that are of 32 dimensions.

Table 6 presents the number of spikes recorded in each of 16 channels and also the a priori number of kinds of spikes found by the preprocessing task. Besides, in the preprocessing task, as PCA components are computed so that different types of spikes are separated, we are going to first consider the projection on the 2 first components of PCA on each channel.

Figure 9 stand for the projection of the spikes of all the events of a selection of channels on the two first components. Whereas Table 6 describes the number of supposed types of spikes and given the preprocessing task, we expect to visualize on Figure 9 the intrinsic structure of the dataset where the number of separated clusters corresponds to those obtained in Table 6. However, it is difficult to visualize in Figure 9 a partition of several clusters in the data for each channel, whereas different clusters for channels 2, 7, and 16 can be observed in Figures 9(a), 9(e), and 9(k); such distinctions cannot be generalized since, on the other channels, it is not possible to visualize a group structure in the projected data. Without the label information of the preprocessing task, nothing enables us to suppose the true existence of different clusters. Furthermore it can be observed in Figure 9(b)—which stands for the projection of data of channel 3 plotted with the labeled spikes elaborated by the preclassification task—that the manual labels give no sense to a partition of the 2 groups of the data.

Consequently, from now, the proposed labels will not be taken into account and the main purpose of this work is to check the relevance of the preprocessing task. This study focuses on channels 2 and 7 whose datasets appear structured.

#### 4.6.1. On Channel 2

**Figure 8:**
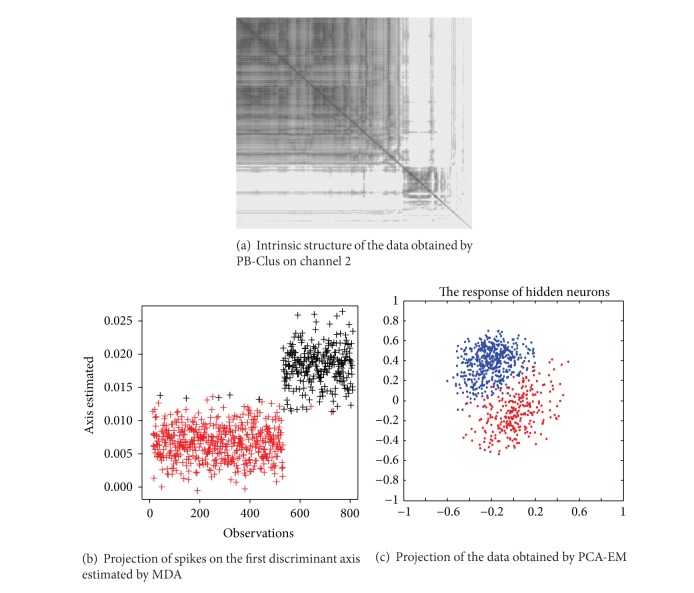
Results obtained by PB-Clus and 2 unsupervised approaches MDA and PCA-EM on channel 2.

**Figure 9:**
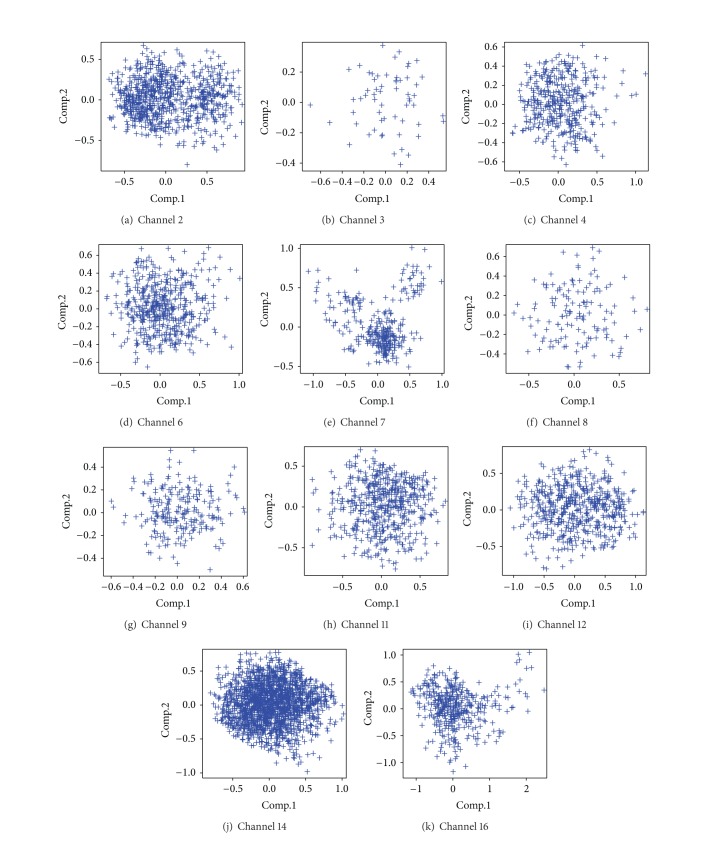
Projection of spikes of each channel on the 2 first components of PCA.

**Figure 10:**
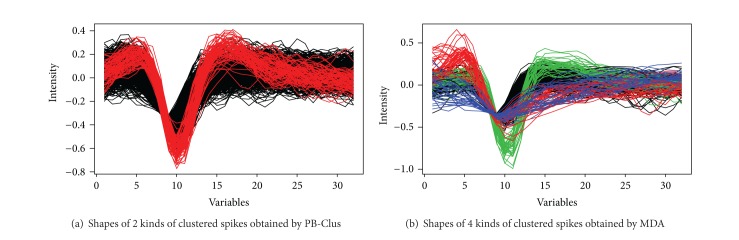
Clustered spikes obtained by PB-Clus in channel 2.

The possible existence of two types of spikes in the axes of PCA in Figure 9(a) is satisfied by both the seriation and the subspace clustering approaches. In Figure 8(a) which represents the rearranged observations obtained by the Algorithm 1, one can observe 2 different blocks, one for each types of spikes in the data. In Figure 8(b) which stands for the projection of the data in the discriminative axes estimated by MDA algorithm, it can be observed that the clusters appear to be well separated compared with those obtained in the PCA axis.  Figure 8(c) illustrates the projection of the data in the discriminative axes estimated by algorithm, it can be observed that the clusters appear to be well separated compared with those obtained in the PCA axis.  Figure 8(c) which stands for the response of the supervised classification by PCA-EM approach has a similar representation of the data as those obtained by their projection in the 2 first principal components of PCA illustrated in Figure 9(a). In addition, Table 7 represents the Fisher index which has been computed for the different approaches previously presented. For the PB-Clus partition, the Fisher index is lower than those obtained by PCA-EM and MDA. It can be explained by the fact that PB-Clus introduces sparsity in the data, which produces smaller clusters that are more compact than those produced by PCA. Besides, the Fisher index for the MDA approach is equal to 588.7, which is equivalent to the result obtained by the PCA-EM classification (*F*_index_ = 585.0) and lower than the PCA's one (*F*_index_ = 864.5). Finally, to check the validity of the partition obtained by both methods, a cross-validation on the *k*-means results obtained by the work of [25] has been made. The contingency table and the clustering accuracy are presented in Table 8 and for each approach, it can be noted that 99% of the labeled data match with the PCA-EM labels. Consequently, it seems that, in channel 2, there are 2 different kinds of spikes and their respective shape obtained by both PB-Clus and MDA approaches is detailed in Figure 10.

#### 4.6.2. On Channel 7

**Figure 11:**
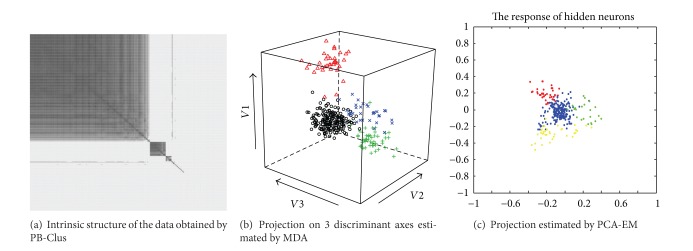
Results obtained by unsupervised approach PB-Clus and 2 supervised approaches MDA and PCA-EM on channel 7.

According to Figure 9(e), it can be observed on the first two components of PCA that there are at least 3 different groups of spikes. This remark is satisfied by the seriation approach, since Figure 11(a) which represents the intrinsic structure obtained by PB-Clus stresses 3 different kinds of spikes. In the same way, 3 components have been selected by using the Bayesian information criterion (BIC) for the mixture model in the case of PCA-EM, whereas both the preprocessing task and MDA, with the computation of BIC, have found 4 types of spikes.  Figure 11(b) represents the projection of the clustered data on the 3 discriminant axes estimated by PCA-EM. In addition, since the *k*-means approach is based on the results of the preprocessing task, the prediction of the class membership of this dataset is made amongst 4 classes as can be seen in Figure 11(c).

Since the number of clusters varies between the different methods, data have been modeled by mixture models with 3 and then 4 components for both PCA-EM and MDA approaches, in order to be able to compare all the approaches. In Table 9 the Fisher index has been computed for the different cases. As expected, this criterion is much lower in the case of PB-Clus since it includes parsimony in the data whereas the ones obtained for MDA or PCA-EM remain high for a mixture of 3 components. Finally, the contingency table and the clustering accuracy are presented in Table 10. It can be observed that for the first case, PB-Clus detects the types 1, 2, and 4 of spikes whereas the 3*rd*⁡ type of spike is mixed with the first one. Furthermore, the classification rate reaches 91% on the spikes retained by PB-Clus when 40% of the data are ousted because of a high level of sparsity. In the second case, the partition obtained by MDA is comparable to these obtained by the PCA-EM classification except for type 1 which is mainly spread on type 3.

**Figure 12:**
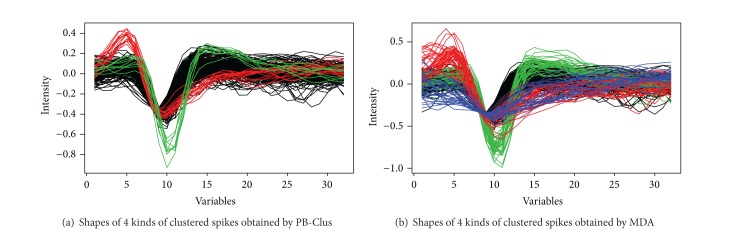
Clustered spikes obtained by PB-Clus and MDA for the channel 7.

Finally, Figures 12(a) and 12(b) show the different spikes clustered by the PB-Clus and MDA algorithms. The difference between the two approaches is clearly seen on the 3*rd*⁡ type of spikes (blue in Figure 12) which is detected by MDA whereas it is not by PB-Clus. This could be explained by the weak dissimilarity between the shape of the 1st and the 3*rd*⁡ type of spikes (resp., black and blue in Figure 12) which is not taken into account by the measure of similarity, the euclidean distance, used in PB-Clus. Different measures of similarity have been tried on PB-Clus such as Spearman correlation or maximum distances but have not brought any more information or any improvement for the visualization.

To conclude, given these results, the existence of 4 different types of clusters does not seem really relevant since some types of spikes, in particular types 3 and 4, are often mixed with the first type in both PB-Clus and MDA approaches. Consequently, either the preprocessing task is biased since the different types of spikes do not really exist or the 32 dimensions of the studied spikes are not sufficient to discriminate the 4 different types of spikes.

## 5. Conclusion

Controlled numerical experiments using spike and noise data extracted from neural recordings indicate significant improvements in detection and classification accuracy compared with amplitude and linear template-based spike sorting techniques. Algorithm 1 makes it possible to visualize subsets of spike data and their dependencies. With this intention, we proposed a family of embedded parsimonious matrices of different levels of parsimony whose level is directly determined by the number of common neighbors between pairs of observations. This is an effective tool for the analysis of data, which offers better results visually than the traditional clustering methods, in particular when the data are noisy or imbalanced or when the groups are superposed.

Moreover, this parsimonious approach facilitates the interpretation of the data and offers a quality of partitioning comparable with the *k*-means method with the advantage of not posing any assumption about the number of clusters. In addition, choosing a level of parsimony in the data corresponds to seeking explicative subsets of a structure. This new point of view can be connected with an approach by levels of density, commonly called* level sets*, which was initially approached by Hartigan [47] and then by Nolan [48]. A comparison of these two approaches and the search for a theoretical bond are part of our research tasks in progress.

## Figures and Tables

**Algorithm 1:**
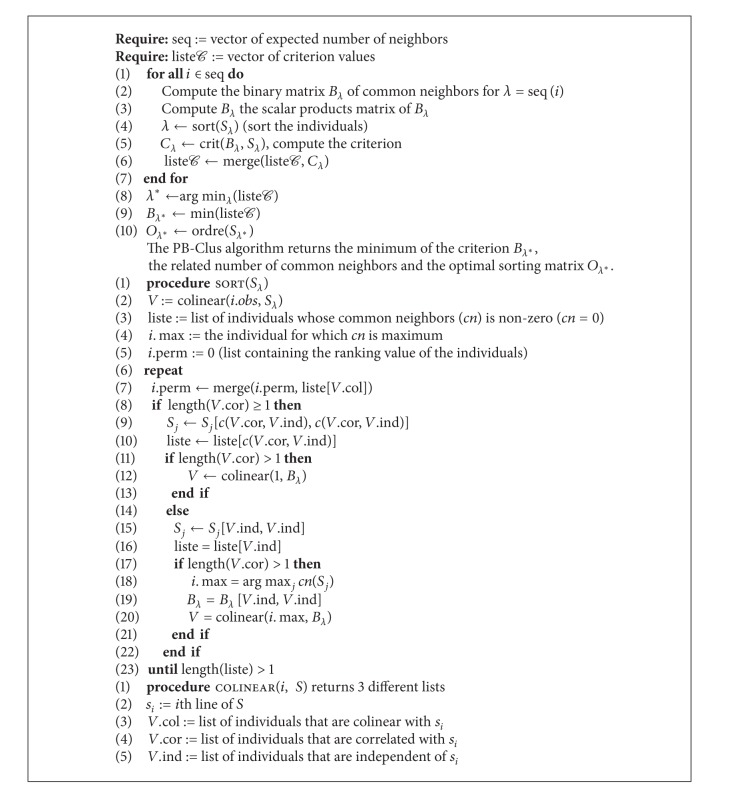
PB-Clus algorithm.

**Table 1:** Criteria of arrangement used within the framework of the clustering one-mode.

Type	Criterion to optimize depending on the dissimilarity matrix *D* = {*d*_*ij*_}_*i*,*j*∈{1,…,*n*}_
Structural criteria	*C*_1_ = ∑_*i*=1_^*n*^‍∑_*j*=1_^*n*^‍*d*_*ij*_ | *i* − *j*|^2^
	*C*_2_ = ∑_*i*=1_^*n*^‍∑_*j*=1_^*n*^‍(*d*_*ij*_ − *α* | *i* − *j*|^2^)
	*C*_3_ = ∑_*i*=1_^*n*−2^‍∑_*j*=*i*+1_^*n*−1^‍∑_*k*=*j*+1_^*n*^‍(*d*_*ij*_ − *α* | *i* − *j*|^2^)
	*C*_4_ = ∑_1≤*i*<*j*<*k*≤*n*_‍*f*(*d*_*ik*_, *d*_*ij*_) + ∑_1≤*i*<*j*<*k*≤*n*_‍*f*(*d*_*kj*_, *d*_*ij*_) with
	*f*(*x*, *y*) = sign⁡(*x* − *y*)
	*f*(*x*, *y*) = = |*x* − *y* | sign⁡(*x* − *y*)
	*f*(*x*, *y*) = *I*_*x*>*y*_
	*f*(*x*, *y*) = |*x* − *y* | *I*_*x*>*y*_
	*C*_5_ = ∑_*j*=*i*+1_^*n*−1^‍∑_*k*=*j*+1_^*n*^‍ | *d*_*ij*_ − *d*_*i*,*j*+1_|

Similarity criteria	*C*_6_ = ∑_*i*,*j*=1_^*n*^‍*d*_*ij*_(*d*_*i*,*j*−1_ + *d*_*i*,*j*+1_ + *d*_*i*+1,*j*_ + *d*_*i*−1,*j*_)
	*C*_7_ = ∑_*i*,*j*=1_^*n*^‍*f*_*ij*_ with
	*f*_*i*,*j*_ = ∑_*k*=max⁡(1,*i*−1)_^min⁡(*n*,*i*+1)^‍∑_*l*=max⁡(*i*,*j*−1)_^min⁡(*n*,*j*+1)^‍(*d*_*ij*_ − *d*_*kl*_)^2^
	*f*_*i*,*j*_ = ∑_*k*=max⁡(1,*i*−1)_^min⁡(*n*,*i*+1)^‍(*d*_*ij*_ − *d*_*kj*_)^2^ + ∑_*l*=max⁡(*i*,*j*−1)_^min⁡(*n*,*j*+1)^‍(*d*_*ij*_ − *d*_*il*_)^2^

**Table 2:** An example of calculation of the *C*_*λ*_*m*__ criterion calculated from the matrix of the introductory example cf Figure 1(a).

Matrix of common neighbors	$B=\begin{array}{cccccc} 2 & 0 & 0 & 3 & 0 & 0\\ 0 & 1 & 0 & 0 & 0 & 0\\ 0 & 0 & 3 & 0 & 3 & 3\\ 2 & 0 & 0 & 3 & 0 & 0\\ 0 & 0 & 3 & 0 & 3 & 3\\ 0 & 0 & 3 & 0 & 3 & 3 \end{array}$		

Parsimony level	*λ* ≥ 1	*λ* ≥ 2	*λ* ≥ 3

Binary matrices of common neighbors	$B=\begin{array}{cccccc} 1 & 0 & 0 & 1 & 0 & 0\\ 0 & 1 & 0 & 0 & 0 & 0\\ 0 & 0 & 1 & 0 & 1 & 1\\ 1 & 0 & 0 & 1 & 0 & 0\\ 0 & 0 & 1 & 0 & 1 & 1\\ 0 & 0 & 1 & 0 & 1 & 1 \end{array}$	$B=\begin{array}{cccccc} 1 & 0 & 0 & 1 & 0 & 0\\ 0 & 0 & 0 & 0 & 0 & 0\\ 0 & 0 & 1 & 0 & 1 & 1\\ 1 & 0 & 0 & 1 & 0 & 0\\ 0 & 0 & 1 & 0 & 1 & 1\\ 0 & 0 & 1 & 0 & 1 & 1 \end{array}$	$B=\begin{array}{cccccc} 1 & 0 & 0 & 1 & 0 & 0\\ 0 & 0 & 0 & 0 & 0 & 0\\ 0 & 0 & 1 & 0 & 1 & 1\\ 1 & 0 & 0 & 0 & 0 & 0\\ 0 & 0 & 1 & 0 & 1 & 1\\ 0 & 0 & 1 & 0 & 1 & 1 \end{array}$

∑_*i*=1_^6^‍∑_*j*=1_^5^‍ | *b*_*i*,*j*_^*λ*_*m*_^ − *b*_*i*,*j*+1_^*λ*_*m*_^|	17	15	9
(calculus per line)	(3 + 2 + 3 + 3 + 3 + 3)	(3 + 0 + 3 + 3 + 3 + 3)	(0 + 0 + 3 + 0 + 3 + 3)

Sorted binary matrices	$B_{1}^{\text{sor}}=\begin{array}{cccccc} 1 & 1 & 0 & 0 & 0 & 0\\ 1 & 1 & 0 & 0 & 0 & 0\\ 0 & 0 & 1 & 1 & 1 & 0\\ 0 & 0 & 1 & 1 & 1 & 0\\ 0 & 0 & 1 & 1 & 1 & 0\\ 0 & 0 & 0 & 0 & 0 & 1 \end{array}$	$B_{2}^{\text{sor}}=\begin{array}{cccccc} 1 & 1 & 0 & 0 & 0 & 0\\ 1 & 1 & 0 & 0 & 0 & 0\\ 0 & 0 & 1 & 1 & 1 & 0\\ 0 & 0 & 1 & 1 & 1 & 0\\ 0 & 0 & 1 & 1 & 1 & 0\\ 0 & 0 & 0 & 0 & 0 & 0 \end{array}$	$B_{3}^{\text{sor}}=\begin{array}{cccccc} 1 & 1 & 1 & 0 & 0 & 0\\ 1 & 1 & 1 & 0 & 0 & 0\\ 1 & 1 & 1 & 0 & 0 & 0\\ 0 & 0 & 0 & 0 & 0 & 0\\ 0 & 0 & 0 & 0 & 0 & 0\\ 0 & 0 & 0 & 0 & 0 & 0 \end{array}$

∑_*i*=1_^6^‍∑_*j*=1_^5^‍ | (*b*_*i*,*j*_^*λ*_*m*_^)_sort_ − (*b*_*i*,*j*+1_^*λ*_*m*_^)_sort_|	9	85	3
(calculus per line)	(1 + 1 + 2 + 2 + 2 + 1)	(1 + 1 + 2 + 2 + 2 + 0)	(1 + 1 + 11 + 0 + 0 + 0)

Criterion *C*_*λ*_*m*__	*C*_*λ*_*m*_≥1_ = 9/17 = 1.89	*C*_*λ*_*m*_≥2_ = 8/15 = 1.88	*C*_*λ*_*m*_≥1_ = 3/9 = 3.03

**Table 3:** Influence of the degree of covering of the clusters on the structure detection.

Degree of covering (en%)	0	6.7	13.3	20.0	26.6	33.3	40.0	46.6	53.30	73.3	100
*x*	0.30	0.28	0.26	0.24	0.22	0.10	0.18	0.16	0.14	0.08	0
*y*	0.225	0.21	0.195	0.18	0.165	0.15	0.135	0.120	0.09	0.06	0

Degree of parsimony	5	6	9	35	33	34	35	35	35	35	34
% of evinced values	0.00	0.00	0.00	0.26	0.23	0.34	0.37	0.35	0.39	0.35	0.43
Value of *C*_*λ*_	1.95	2.01	2.42	2.64	2.90	2.82	3.34	3.29	3.32	3.54	3.65

Classification rate	0.99	0.99	0.99	0.99	0.95	0.90	0.86	0.60	0.49	0.44	0.39

**Table 4:** Comparison of 3 methods of seriation, PB-Clus, HC, and Chen approach according to Moore and Neumann criteria on the data benchmarks.

Method	PB-Clus seriation		HC seriation		Chen seriation	
Criterion	Moore	Neumann	Moore	Neumann	Moore	Neumann
Dataset						
Iris	1 371.2	471.1	31 728.8	10 893.1	19 357.8	7 304.0
Townships	244.5	91.8	1 109.9	441.5	849.0	342.0
Ruspini	1 290.1	442.2	8 724.9	3 036.4	6 503.7	2 277.1
Faithful	2 634.1	889.4	34 045.5	11 503.5	23 390.0	9 894.2
Geysers	2 514.9	850.4	68 205.3	2 302.1	12 866.8	4 501.4

**(a):** 

Fisher Iris								Township				
Known classes	Clusters PB-Clus			Known classes	Clusters *k*-means			Known classes	Clusters PB-Clus			
	1	2	3		1	2	3		1	2	3	4
Setosa	50	0	0	Setosa	50	0	0	Urban cities	8	0	0	0
Versicolor	0	50	0	Versicolor	0	49	1	Transitions	0	4	0	0
Virginica	0	17	33	Virginica	0	13	37	Country towns	0	0	2	0
								Unclassified	0	1	0	1

Classification rate =0.88				Classification rate = 0.90				Classification rate = 0.94				

**(b):** 

Ruspini					Faithful			Geysers			
Clusters *k*-means	Clusters PB-Clus				Clusters *k*-means	Clusters PB- Clus		Clusters *k*-means	Clusters PB-Clus		
	1	2	3	4		1	2		1	2	3
Group 1	50	0	0	0	Group 1	168	4	Group 1	88	2	7
Group 2	0	35	0	0	Group 2	0	100	Group 2	0	105	0
Group 3	0	0	15	0				Group 3	0	0	97
Group 4	0	0	0	20							

Classification rate = 1.00					Classification rate = 0.98			Classification rate = 0.97			

**Table 6:** Number and type of spikes recorded in the 16 channels.

Channel	1	2	3	4	5	6	7	8	9	10	11	12	13	14	15	16
Number of spikes	—	799	60	405	727	489	300	229	475	224	533	538	21	1833	1491	421
Types of spikes	—	2	2	2	1	2	4	1	1	2	2	2	1	2	1	4

**Table 7:** Fisher index computed in channel 2 for PCA-EM, PB-Clus, and MDA.

Methods	PCA-EM	PB-Clus	MDA
*F*_index_	864.5	210.1	588.7

**Table 8:** Contingency tables for PB-Clus and MDA partitions with *k*-means classification in channel 2.

PB-Clus			MDA		
*k*-means classes	Clusters		*k*-means classes	Clusters	
	1	2		1	2
Type 1	214	0	Type 1	515	2
Type 2	0	79	Type 2	5	277

Classification rate = 100%			Classification rate = 99.12%		

**Table 9:** Fisher index computed in channel 7 for PCA-EM, PB-Clus, and MDA.

Methods	PCA-EM	PB-Clus	MDA
*F*_index_ for 3 clusters	614.1	287.9	483.6
*F*_index_ for 4 clusters	392.2	—	324.1

**Table 10:** Contingency tables for PB-Clus and MDA partitions with *k*-means classification for the channel 7.

PB-Clus				MDA				
*k*-means classes	Clusters			*k*-means classes	Clusters			
	1	2	3		1	2	3	4
Type 1	183	11	0	Type 1	181	0	17	6
Type 2	0	0	7	Type 2	1	36	0	0
Type 3	5	0	0	Type 3	0	0	33	4
Type 4	1	5	1	Type 4	0	0	0	22

Classification rate = 91%				Classification rate = 90.1%				
